# Sarcopenia in patients with chronic kidney disease not yet on dialysis: Analysis of the prevalence and associated factors

**DOI:** 10.1371/journal.pone.0176230

**Published:** 2017-04-27

**Authors:** Viviane Angelina de Souza, Dílmerson Oliveira, Sérgio Ribeiro Barbosa, José Otávio do Amaral Corrêa, Fernando Antônio Basile Colugnati, Henrique Novais Mansur, Natália Maria da Silva Fernandes, Marcus Gomes Bastos

**Affiliations:** 1 Department of Clinical Medicine, School of Medicine, Federal University of Juiz de Fora, Juiz de Fora, Brazil; 2 Department of Anatomy, School of Physical Education, UNIFAMINAS, Muriaé, Brazil; 3 Department of Pharmaceutical Sciences, School of Pharmacy, Federal University of Juiz de Fora, Juiz de Fora, Brazil; 4 Internship Department, School of Medicine, Federal University of Juiz de Fora, Juiz de Fora, Brazil; 5 Department of Education, School of Physical Education, Federal Institute of Education, Science and Technology – Southeast of Minas Gerais - Campus Rio Pomba, Rio Pomba, Brazil; The University of Tokyo, JAPAN

## Abstract

**Introduction:**

Sarcopenia is a chronic condition that is associated with aging and characterized by a reduction of muscle mass, strength, and function. Sarcopenia is prevalent in patients with chronic kidney disease (CKD) and associated with increased morbidity and mortality, as well as cardiovascular complications.

**Objectives:**

To investigate the prevalence of sarcopenia in patients with CKD not yet on dialysis and its correlation with clinical and laboratory variables and inflammatory markers.

**Methods:**

A total of 100 patients of both sexes aged over 18 were evaluated. Sarcopenia was defined using the criteria of the European Working Group on Sarcopenia in Older People (EWGSOP) and of the Foundation for the National Institutes of Health (FNIH) Sarcopenia Project. Sociodemographic and clinical data, activities of daily living, functional capacity, and physical activity were also evaluated. Inflammation was assessed by the serum levels of high-sensitivity C-reactive protein (hsCRP) and interleukin (IL) 4 and 6.

**Results:**

The prevalence of sarcopenia was 11.9% and 28.7% using the EWGSOP and FNIH criteria, respectively. Sarcopenia was more prevalent in the more advanced stages of CKD (34.5% in stages 2 and 3A; and 65.5% in stages 3B, 4, and 5) and associated with worse performance in activities of daily living (p = 0.049), lower walking speeds (p < 0.001), and higher body mass indexes (BMIs) (p = 0.001) in the non-adjusted model. In addition, patients with sarcopenia had lower functional capacity (p = 0.012) and higher prevalence of physical inactivity (p = 0.041) compared with patients without sarcopenia. After adjustment for confounding variables, sarcopenia was still significantly correlated with walking speed (p = 0.004) and BMI (p = 0.002). HsCRP levels were inversely correlated with appendicular lean mass adjusted for BMI (p = 0.007) and were also positively associated with BMI (p = 0.001). IL4 levels were positively correlated with walking speed (p = 0.007) and lean mass in the lower limbs (p = 0.022).

**Conclusions:**

Sarcopenia is common in patients with CKD, particularly in the most advanced stages of the disease. We observed an association between the levels of inflammatory markers and peripheral lean body mass, physical performance, and BMI. This association between sarcopenia and modifiable factors highlights the importance of early diagnosis and the implementation of therapeutic measures to minimize adverse outcomes in patients with CKD not yet on dialysis.

## Introduction

The percentage of older people in the population has increased, which emphasizes the need to understand the degenerative processes that occur with advancing age [1]. One of these processes is sarcopenia, which is defined as the progressive, age-related loss of muscle mass combined with a decline in muscle function and physical performance. Sarcopenia may have deleterious effects on the health of older people [2].

Sarcopenia is an impaired health state that is characterized by locomotor disorder, increased risk of falls and fractures, difficulty in performing activities of daily living (ADLs), disability, loss of independence, and increased risk of death [3–5].

The cause of sarcopenia is multifactorial and usually associated with environmental factors, chronic diseases, activation of inflammatory mediators, physical inactivity, mitochondrial abnormalities, loss of neuromuscular junctions, a decrease in the number of satellite cells, and hormonal changes [6].

A high prevalence of sarcopenia has been reported in developed and developing countries, and this disorder affects both healthy individuals and individuals with chronic diseases, including hypertension, diabetes, and chronic kidney disease (CKD) [7–9].

In CKD, sarcopenia may affect approximately 37% of dialysis patients [10]. However, its prevalence in earlier stages of CKD is poorly understood and ranges between 5% and 9% [11]. The loss of muscle mass in this population is correlated with greater morbidity and mortality, particularly due to an increase in cardiovascular complications [12]. Therefore, the early identification of sarcopenia and evaluation of the modifiable factors associated with it are essential.

Data from the literature show that some inflammatory markers such as IL6, CRP and tumor necrosis factor (TNF) alpha were associated with sarcopenia [13], and in patients with CKD, these markers were higher than in the patients with normal renal function and were associated with unfavorable outcomes [14]. However, its use in clinical practice is limited and research is needed to better define its association with sarcopenia.

In recent years, different definitions of sarcopenia have been proposed, and there is no consensus on its characterization. The diagnostic criteria of the European Working Group on Sarcopenia in Older People (EWGSOP) were proposed in 2010 [2] and are widely used in clinical practice and research. However, new definitions for sarcopenia have recently been proposed by the Foundation for the National Institutes of Health (FNIH) Sarcopenia Project [15]

Considering the limited number of studies evaluating sarcopenia in patients with CKD not yet on dialysis and the potentially unfavorable outcomes associated with this syndrome, the objectives of this study were to determine the prevalence of sarcopenia in patients with CKD on conservative treatment using two diagnostic criteria and examine the correlations between sarcopenia, clinical and laboratory variables, and inflammatory markers. Additionally, we also evaluated the association between inflammatory markers and clinical variables related to sarcopenia.

## Materials and methods

This cross-sectional, convenience sample based study evaluated 100 patients from the CKD outpatient clinic of the HIPERDIA Center of the IMEPEN Foundation. This foundation is a secondary care facility that is specialized in the treatment of patients with hypertension and high risk of cardiovascular disease, diabetes mellitus types I and II with poor metabolic control, and patients with CKD not yet on dialysis. The study was approved by the Ethics and Research Committee of the Federal University of Juiz de Fora. Signed informed consent was obtained from all study participants prior to data collection. Inclusion criteria for study enrollment included being an ambulatory male or female adult who were aged over 18, with CKD on conservative treatment. Contraindications to participate in the study included patients with limited performance of tests necessary for assessment of muscle strength and function due to severe neuropathy, liver disease, stroke sequelae, arthritis, arthrosis, amputations, severe chronic obstructive pulmonary disease, Parkinson's disease, cancer, and HIV/AIDS.

CKD was defined and classified according to the criteria of KDIGO [16], and the estimated glomerular filtration rate (eGFR) was determined using the equation of the CKD Epidemiology Collaboration (CKD-EPI) [17]. Due to the small sample size, the statistical comparisons were made between subjects with eGFR ≥ 45 mL/min/1.73 m^2^ (stages 2 and 3A) and those with < 45 mL/min/1.73m^2^ (stages 3B, 4 and 5).

Systemic hypertension was defined as a systolic blood pressure (BP) equal to or greater than 140 mmHg and a diastolic BP equal to or greater than 90 mmHg or the use of antihypertensive medications [18]. Diabetes mellitus was defined as fasting glycemia greater than or equal to 126 mg/dL or the use of hypoglycemic drugs [19].

The study participants completed a questionnaire that addressed sociodemographic characteristics, ADLs using Lawton’s scale [20], functional capacity using the SF-36 questionnaire [21], and the level of physical activity using the Minnesota questionnaire [22]. A physical examination was also performed to assess BP and body mass index (BMI).

To evaluate muscle mass, the patients were subjected to bone densitometry with full-length acquisition via dual-energy X-ray absorptiometry using a GE Lunar Prodigy Primo device. To determine low muscle mass, the appendicular lean mass index (ALMI) was measured following the criteria established by Baumgartner [23] and advocated for by the EWGSOP [2] using the following formula: appendicular lean mass (ALM)/height^2^. A state of low muscle mass was assigned to cases in which the ALMI values were lower than 7.26 kg/m^2^ for men and lower than 5.5 kg/m^2^ for women. In the definition of sarcopenia proposed by the FNIH, the ALM was divided by BMI. A state of low muscle mass was assigned if the values were lower than 0.789 for men and lower than 0.512 for women [15].

A handgrip was used to evaluate muscle strength, and the cutoff point was < 30 kg for men and < 20 kg for women, according to the EWGSOP criteria. The criteria of the FNIH uses a cutoff point of < 26 kg for men and < 16 kg for women.

Muscle performance was evaluated by calculating walking speed over a distance of 3 meters, and a speed of < 0.8 m/s indicated low performance using both criteria.

The cutoff points for low muscle mass, muscle strength and performance, according to each one of the criteria, are detailed in Table 1.

**Table 1:** FNIH and EWGSOP recommended criteria for sarcopenia.

Criteria	Measure	Cut-point	
		Men	Women
**FNIH**			
Muscle Mass Muscle Strength Muscle performance	ALM divided by BMI Handgrip strength Walking speed	< 0,789 < 26 kg < 0,8 m/s	< 0,512 < 16 kg < 0,8 m/s
**EWGSOP**			
Muscle Mass Muscle Strength Muscle performance	ALM divided by H^2^ Handgrip strength Walking speed	< 7,26 kg/m^2^ < 30 kg < 0,8 m/s	< 0,545 kg/m^2^ < 20 kg < 0,8 m/s

FNIH: Foundation for the National Institutes of Health; EWGSOP: European Working Group for Sarcopenia in Older People; ALM: appendicular lean mass; BMI: body mass index; H^2^: squared height.

Blood samples were collected after 12 hours of fasting for evaluation of blood count, albumin, glucose, total cholesterol and fractions, triglycerides, 25-hydroxyvitamin D, parathyroid hormone, calcium, phosphorus, and creatinine. High-sensitivity C-reactive protein (hsCRP) and interleukin (IL) 6 levels were measured to evaluate inflammation. IL4 was used as an anti-inflammatory marker. HsCRP levels were measured using immunoturbidimetry, and IL4 and IL6 levels were measured using Enzyme-Linked Immunosorbent Assay (ELISA). The urine protein-to-creatinine ratio was determined from a single urine sample per patient, and values > 0.20 mg/g were considered abnormal.

### Statistical analysis

The results were expressed as mean and standard deviation for continuous variables unless otherwise specified. Categorical variables were expressed as percentages.

Sarcopenia was defined using the classification criteria of the FNIH because this classification adjusts the ALM by BMI and not simply by height.

The differences between the groups at baseline were assessed by bivariate analysis using the chi-square test or the t-test, depending on the variables analyzed. Logarithmic transformation was applied to IL4 and IL6 in all analysis, since these data did not present normal distribution. Although hsCRP did not present normal distribution as well, logarithmic transformation was not adequate since it generated negative values for values lower than 1. Median and inter-quartile interval was used to summarize this marker.

Box-plots were used to show the distribution of the inflammatory markers in the two groups. Box corners represented the first (lower corner) and third (upper corner) quartiles, and median represented by the line inside boxes. Whiskers represented lower and upper theoretical limits.

Spearman’s correlation coefficient was used to assess the associations between hsCRP, logarithm (Log) IL 4 and sarcopenia related variables.

The likelihood of sarcopenia given its criteria, adjusted for possible confounding variables, was evaluated using a multivariate logistic regression model with a confidence interval of 95%. A hierarchical approach for model adjustment was adopted using the variables that were significantly associated with sarcopenia in the bivariate analysis. The ALMI variables adjusted for BMI and lean mass in the lower limbs were excluded from this model because of the high collinearity with the sex variable, which interfered with the analysis.

Version 21.0 of the SPSS software was used for data analysis. P-values < 0.05 were considered statistically significant.

## Results

In the population studied, the mean age was 73.59 ± 9.22 years, and 58.4% of the participants were women. There was a predominance of stages 3B (37%) and 4 (29%) of CKD. All patients had hypertension and 53.5% had diabetes.

The prevalence of sarcopenia was 11.9% using the EWGSOP criteria and 28.7% using the FNIH criteria. The prevalence of sarcopenia was 34.5% in stages 2 and 3A of CKD and 65.5% in stages 3B, 4, or 5. When we analyzed the components of sarcopenia separately, we observed that 44% of patients had reduced muscle mass, 9% reduced muscle strength, while 69% of patients had reduced muscle performance. The clinical and sociodemographic characteristics of the total study population and the groups with and without sarcopenia are shown in Table 2.

**Table 2:** Clinical and sociodemographic characteristics of the study groups.

Variables	Total (n = 100)	Sarcopenic (n = 29)	Non sarcopenic (n = 71)	p-value
**Age (years)**	73.59 ± 9.22	78.21 ± 8.46	71.70 ± 8.93	0.001
**Sex (Female/Male)**	59/41	18/11	41/30	0.690
**Education (years)**	3,78 ± 3,47	2.93 ± 2.80	4.13 ± 3.65	0.080
**Activities of daily living**	6.32 ± 1.82	5.76 ± 1.74	6.55 ± 1.81	0.049
**Walking speed (m/s)**	0.82 ± 0.48	0.542 ± 0.18	0.934 ± 0.52	<0.001
**Handgrip (kg)**	27 ± 9.42	24.48 ± 8.74	28.03 ± 9.55	0.079
**Systolic blood pressure (mmHg)**	152 ± 25.74	150.69 ± 22.82	152.54 ± 26.97	0.729
**Diastolic blood pressure (mmHg)**	87 ± 13.49	84.83 ± 12.13	88.17 ± 13.97	0.237
**BMI (kg/m**^**2**^**)**	28.75 ± 5.54	32.04 ± 5.91	27.40 ± 4.81	0.001
**Functional capacity (SF-36)**	60.45 ± 28.48	49.31 ± 25.83	65.0 ± 28.42	0.012
**Physical activity (active/inactive)**	17/83	1/28	16/55	0.021
**ALM in the upper limbs (kg)**	4,64 ±1,23	4.38 ± 1.17	4.74 ± 1.24	0.173
**ALM in the lower limbs (kg)**	13,25 ± 2,75	12.40 ± 2.87	13.60 ± 2.64	0.049
**Total lean mass**	41.85 ± 8.67	40.21 ± 8.07	42.51 ± 8.87	0.215
**ALMI (kg/height**^**2**^**)**	7.188 ± 1.088	7.18 ± 1.22	7.18 ± 1.037	0.981
**ALMI (kg/BMI)**	0.646 ± 0.160	0.531 ± 0.135	0.694 ± 0.146	<0.001

Data are expressed as the mean ± standard deviation, median (minimum-maximum), or n. BMI: body mass index; ALM: appendicular lean mass; ALMI: appendicular lean mass index.

The patients with sarcopenia were older (p = 0.001), showed worse performance in ADLs (p = 0.049), had lower walking speeds (p <0.001), had higher BMIs (p = 0.001), had less lean mass in their lower limbs (p = 0.049), and had less ALM when adjusted for BMI (p < 0.001) in the unadjusted model. In addition, these patients had worse functional capacity (p = 0.012) and a higher prevalence of physical inactivity (p = 0.021).

Moreover, the patients with sarcopenia tended to have higher levels of triglycerides (p = 0.053), significantly lower levels of serum creatinine (p = 0.001), and a non-significantly difference of proteinuria (17.4% vs. 14.1%, p = 0.914) compared with patients without sarcopenia (Table 3).

**Table 3:** Laboratory characteristics of the study groups.

Variables	Total (n = 100)	Sarcopenic (n = 29)	Non sarcopenic (n = 71)	p-value
**Hemoglobin (g%)**	12.40 ± 1.75	12.71 ± 1.40	12.27±1.87	0.202
**Glucose (mg/dL)**	116.54 ± 45.69	120.52 ± 42.11	114.92 ± 47.26	0.563
**Total cholesterol (mg/dL)**	172.56 ± 41.50	177.10 ± 41.02	170.70 ± 41.84	0.485
**HDL (mg/dL)**	42.20 ± 10.29	40.48 ± 9.39	42.90 ± 10.63	0.266
**LDL (mg/dL)**	98.21 ± 38.62	100.62 ± 32.33	99.23 ± 36.59	0.851
**Triglycerides (mg/dL)**	154.07 ± 91.85	181.86 ± 111.95	142.72 ± 80.42	0.053
**Albumin (g/dL)**	4.01 ± 0.31	3.97 ± 0.30	4.03 ± 0.32	0.455
**Calcium (mg/dL)**	9.01 ± 0.64	9.08 ± 0.66	8.98 ± 0.63	0.483
**Phosphorous (mg/dL)**	3.93 ± 1.07	3.72 ± 0.56	4.02 ± 1.21	0.094
**25(OH)D (ng/dL)**	28.67 ± 10.06	29.59 ± 13.61	28.30 ± 8.28	0.637
**PTH (pg/ml)**	664.84 (7.36–672.20)	162.6 (7.36–657.50)	161.7 (36.20–672.20)	0.470
**Creatinine (mg/dL)**	5.24 (1.00–6.24)	1.50 (1.00–6.24)	2.00 (1.00–6.24)	0.001
**ACR (mg/g of creatinine)**	0.29 (0.04–4.24)	0.19 (0.05–1.41)	0,30 (0.04–4.24)	0.215
**eGFR (ml/min/1.73 m**^**2**^**)**	35,96 ± 16,01	38,88 ± 12,85	34.76 ± 17.08	0.246
**hsCRP (mg/L)**	3.32 (0.05–41.12)	5.40 (0.05–29.27)	2.70 (0.05–41.12)	0.075
**LogIL6 (pg/ml)**	7.05 (6.15–7.89)	6.96 (6.35–7.81)	7.06 (6.15–7.89)	0.593
**LogIL4 (pg/ml)**	6.80 (5.54–7.84)	6.72 (6.15–7.84)	6.84 (5.54–7.81)	0.071

Data are expressed as the mean ± standard deviation or median (minimum-maximum). t-test, x^2^, p < 0.05. 25(OH)D: 25-hydroxyvitamin D; PTH: parathyroid hormone; ACR: albumin/creatinine ratio; eGFR: estimated glomerular filtration rate; hsCRP: high-sensitivity C-reactive protein; LogIL: log of interleukin.

**Fig 1:**
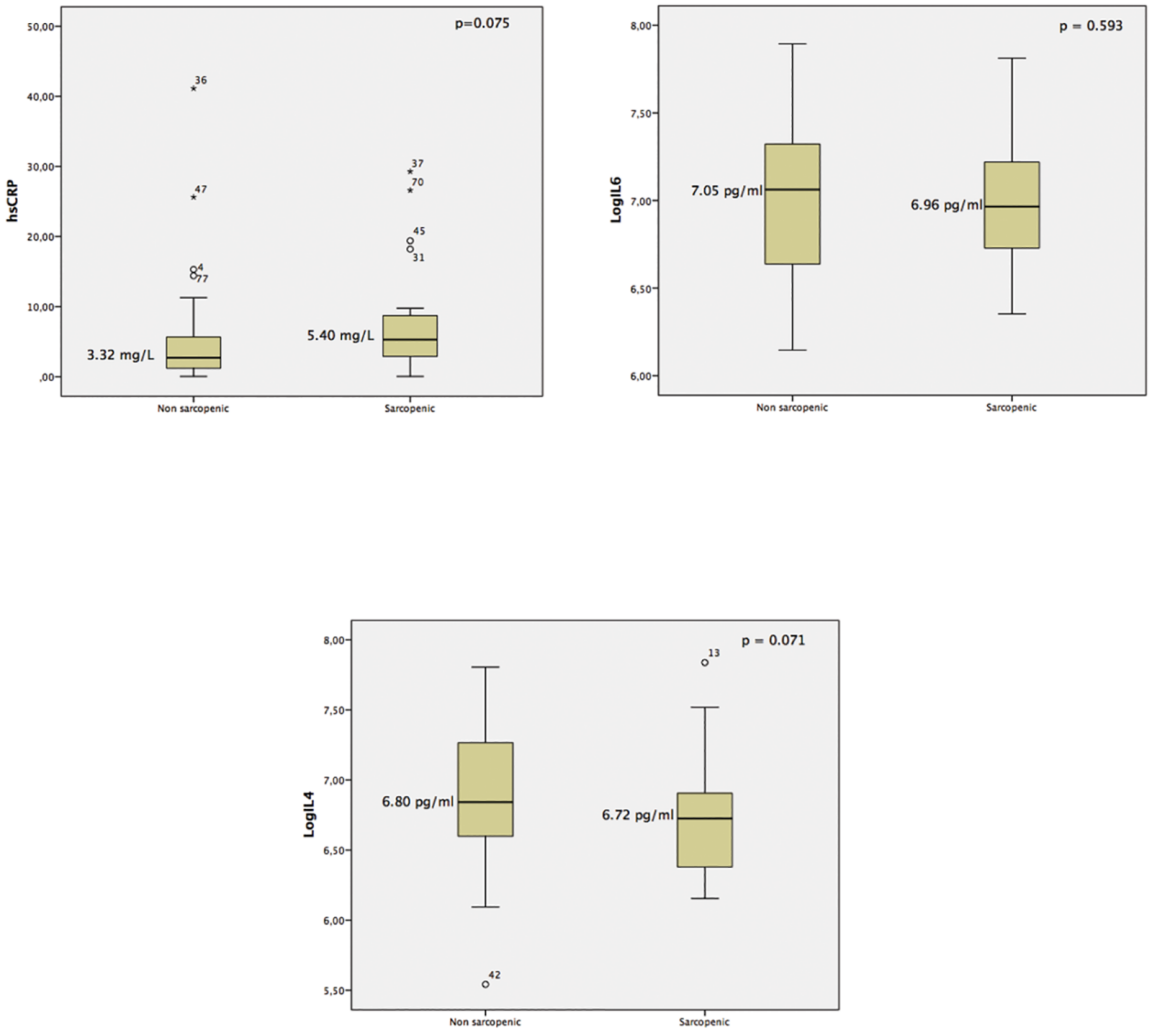
Boxplot of the levels of high-sensitivity C-reactive protein (A) and log for Interleukin 6 (B) and 4 (C) in patients with and without sarcopenia. Box-plots are used to present inflammatory marker distributions between groups. Box corners represent the first (lower corner) and third (upper corner) quartiles, and median represented by the line inside boxes. Whiskers represent lower and upper theoretical limits. hsCRP: high-sensitivity C-reactive protein; LogIL: log of interleukin. p < 0.05.

Regarding the inflammatory markers, we observed that patients with sarcopenia showed a trend of higher levels of hsCRP and lower levels of the anti-inflammatory cytokine IL4. However, there was no difference in the levels of IL6 between the groups (Table 3 and Fig 1).

**Fig 2:**
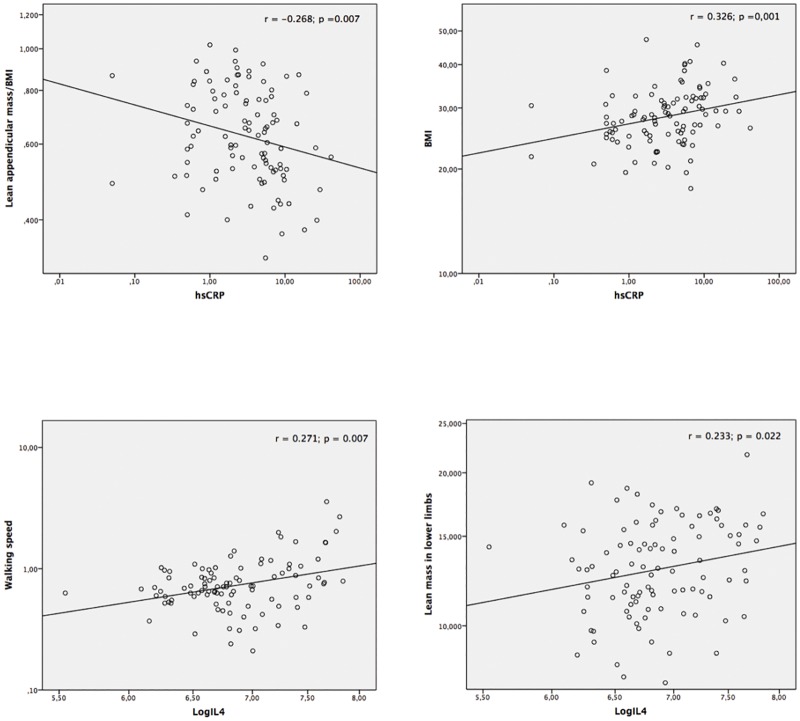
Scatter plot between high-sensitivity C-reactive protein (A and B) and log for interleukin 4 (C and D) and clinical variables. Spearman’s correlation coefficient, p < 0.05. BMI: body mass index; hsCRP: high-sensitivity C-reactive protein; LogIL: log of interleukin.

The hsCRP levels were inversely associated with ALM adjusted for BMI (r = –0.268, p = 0.007), and they were also positively correlated with BMI (r = 0.326; p = 0.001). IL4 levels showed a positive correlation with walking speed (r = 0.271, p = 0.007) and lean mass in the lower limbs (r = 0.233, p = 0.022) (Fig 2).

In the multivariate logistic regression model, after adjusting for age, sex, ADLs (Lawton’s scale), walking speed, BMI, functional capacity (SF-36 questionnaire), level of physical activity (Minnesota questionnaire), and estimated glomerular filtration rate, sarcopenia was still significantly correlated with walking speed (odds ratio [OR]: 0.007; 95% confidence interval [CI]: 0.000–0.212; p = 0.004) and BMI (OR: 1.256; 95% CI: 1.088–1.451; p = 0.002) (Table 4).

**Table 4:** Odds ratio for sarcopenia adjusted for confounding variables.

Variable	Odds ratio	95% CI		p-value
		Lower	Upper	
**Age**	1.059	0.975	1.151	0.176
**Female**	2.329	0.598	9.065	0.223
**Activities of daily living**	0.908	0.647	1.274	0.577
**Walking speed (m/s)**	0.007	0.000	0.212	0.004
**Body mass index (kg/m**^**2**^**)**	1.256	1.088	1.451	0.002
**Functional capacity**	0.984	0.964	1.005	0.133
**Physical activity**	0.570	0.108	3.021	0.509
**Glomerular filtration rate (ml/min/1.73 m**^**2**^**)**	1.015	0.956	1.078	0.625

The model included the variables age, sex, activities of daily living index (Lawton’s scale), walking speed, body mass index,functional capacity (SF-36 questionnaire), level of physical activity(Minnesota questionnaire) and estimated glomerular filtration rate. P < 0.05, R square = 0.567.

## Discussion

The main findings of our study were the demonstration that in patients with CKD not yet on dialysis, the prevalence of sarcopenia varied according to the diagnostic criteria used, and the results suggest that the EWGSOP criteria underestimated this prevalence in the studied population. We also observed a relationship between increased prevalence of sarcopenia and worsening of renal function. The inflammatory markers analyzed were associated with sarcopenia and associated clinical variables as well, mainly modifiable factors such as walking speed and BMI.

Sarcopenia, which is characterized by the progressive, age-related loss of muscle mass coupled with a decline in muscle function and physical performance [2], is gaining importance in the academic community because of its unfavorable outcomes. In addition to its association with the aging process, sarcopenia is also associated with chronic diseases, including CKD. In CKD, the majority of the studies on sarcopenia have evaluated hemodialysis patients. However, few studies have investigated the early stages of CKD. The definition of sarcopenia is still not fully established in the literature, and different diagnostic criteria are used in research and clinical practice.

In this study, we aimed to evaluate the prevalence of sarcopenia in patients with CKD not yet on dialysis using two diagnostic criteria. According to the EWGSOP criteria, in which the definition of low muscle mass is adjusted to the squared height, the prevalence of sarcopenia in the general population is approximately 30% and might reach 50% in individuals aged over 80 [2]. Using these criteria, Pereira et al. reported a prevalence of 5.9% in patients with CKD on conservative treatment [11]. However, the EWGSOP criteria were based on statistical characteristics of the lean mass from the elderly in New Mexico [23], and in this way, as a limitation, they may not be generalized. Furthermore, they lack a relationship to important outcomes such as muscle strength and function. On the other hand, the FNIH criteria were originated from compiled data of multiple cohorts with large representative samples of a wide variety of the elderly community. Han et al., using these criteria, found a prevalence of 7% in the general population, which reached 14.8% when individuals aged over 60 were included. However, in this study only muscle mass was taken into account to characterize sarcopenia [24]. Using the EWGSOP criteria, we observed a prevalence of sarcopenia in our patients with CKD of 11.9%, and, according to the FNIH criteria, the prevalence reached 28.7%, suggesting that the EWGSOP criteria underestimated the prevalence of sarcopenia in patients with CKD not yet on dialysis. The difference observed in the prevalence may be due to the classification of low muscle mass between both criteria. To the best of our knowledge, this study is the first to evaluate sarcopenia using the FNIH criteria in this population of patients.

In our point of view, the criteria proposed by the FNIH are the most appropriate to be used in clinical research not only for being more representative of the general elderly population, but also for being the first criteria that observed an association with walking speed, a clinical outcome directly associated with muscular disability. Sensitivity analysis indicated that obesity influenced the relationship between muscle mass and strength, which suggests that the adjustment of the muscle mass by BMI would be more appropriate [15].

Several studies have reported correlations between the increased prevalence of sarcopenia and worsening kidney function, and there is evidence of an association between sarcopenia and albuminuria [24–26]. Herein, we observed a higher prevalence of sarcopenia among individuals with CKD in stages 3B, 4, and 5. However, this disorder also developed in the early stages of CKD, indicating the need for early diagnosis of sarcopenia in renal patients in order to establish measures to prevent its progression and its related complications. We observed a higher prevalence of proteinuria in patients with sarcopenia, but the increase was not significant.

In our study, CKD patients with sarcopenia had worse physical performance, which was reflected by low walking speed, worse performance in ADLs, less functional capacity, and a higher prevalence of physical inactivity compared with patients without sarcopenia. Published data have reported an association between sarcopenia and unfavorable outcomes, including physical inactivity, poor functional capacity, increased risk of falls, and death [3,4, 27–29]. In a recent systematic review, Hirai et al. found that sarcopenia and physical inactivity progressed synergistically in patients with CKD and were predictors of mortality in this population [30].

In patients with CKD and obesity, the production of inflammatory mediators by adipose tissue is associated with an increased prevalence of cardiovascular complications and increased mortality [31]. Thus, the association between sarcopenia and obesity, also known as sarcopenic obesity, seems to contribute significantly to the occurrence of unfavorable outcomes in this population, including decreased physical function [30, 32]. As already mentioned, obesity can influence muscle strength, probably due to lipid infiltration in muscle tissues, impairing the incorporation of amino acids and reducing the synthesis of muscle proteins. However, in patients with renal disease on dialysis, a correlation between higher BMI and better survival, which is termed reverse epidemiology, has been reported [33–35]. BMI may underestimate the prevalence of sarcopenia, as this index does not differentiate fat mass from lean muscle mass. Thus, groups with higher BMI include individuals with obesity who maintained muscle mass, which is a better overall health marker. This hypothesis could explain the "protective effects" of obesity in populations with CKD and end-stage renal disease (ESRD) [33,36], meaning that the highest BMI could be related not to fat mass but to the presence of a greater amount of lean mass, which could actually "protect" these patients. Honda et al. observed a higher prevalence of obesity and sarcopenic obesity in patients with ESRD, and after adjusting for confounding variables, elevated BMI was associated with longer survival [31]. Our results showed that sarcopenic patients with CKD had higher BMI values than patients without sarcopenia, and this association persisted after adjustment for confounding variables. In addition, we observed an association between BMI and inflammation. However, this finding may have been influenced by the higher prevalence of physical inactivity among sarcopenic patients. These results indicate the need for further studies to evaluate the occurrence of sarcopenic obesity in patients with CKD to elucidate the paradigm of the beneficial effects of a higher BMI in patients with CKD in renal replacement therapy.

We have shown evidences of the association between sarcopenia and some clinical variables with inflammatory markers. Our results suggested that there was a trend towards higher levels of hsCRP and lower levels of the anti-inflammatory cytokine IL4 in patients with sarcopenia, however, there was no difference in the levels of IL6 between the groups. We also observed inverse associations between hsCRP levels and peripheral muscle mass. IL4 was positively associated with walking speed and lean mass in the lower limbs. It is well described in the literature that the oxidative stress and increased levels of inflammatory mediators are two possible mechanisms contributing to the pathogenesis of sarcopenia [37]. Increased levels of inflammatory cytokines such as IL6 and TNF- alpha, as well as hsCRP, were associated with sarcopenia in the general population and patients with ESRD [31,38]. Batsis et al. evaluated the prevalence of sarcopenia in the general population and observed an association between ALM adjusted for BMI and hsCRP levels [39]. In a recent systematic review and meta-analysys, Bano et al concluded that sarcopenia seems to be associated with elevated serum CRP levels, but no association was found with IL6 when compared to controls [40]. However, prospective studies with larger sample sizes involving the analysis of various inflammatory mediators will be necessary to elucidate the role of inflammation in sarcopenic CKD patients not yet on dialysis.

Our study has some limitations. The small sample size and cross-sectional nature of this study limited the establishment of a causal relationship between sarcopenia and patients with CKD not yet on dialysis. Furthermore, the evaluation of eGFR using the creatinine based CKD-EPI equation might have been influenced by the muscle consumption associated with sarcopenia. The exclusion criteria were many and very broad, which could have probably lead to a selection bias and to an underestimation of the prevalence of sarcopenia. Finally, considering the regression model, we emphasize that the findings related to walking speed and BMI should be interpreted with caution, since they are variables involved in the sarcopenia classification criteria.

## Conclusions

In summary, sarcopenia was common in patients with CKD not yet on dialysis in this study, particularly during the more advanced stages and the EWGSOP criteria underestimated its prevalence. We observed correlations between inflammatory markers with ALM adjusted by BMI, physical performance and BMI. The association of sarcopenia with modifiable factors highlights the importance of early diagnosis and implementation of therapeutic strategies to minimize adverse outcomes in patients with CKD not yet on dialysis. The prevalence of sarcopenia is expected to increase as the population ages, which calls for the development of prospective studies with larger sample sizes to elucidate the correlation between sarcopenia and CKD.

## Supporting information

S1 File (XLS)
